# Development of a new chromatographic method for the determination of bakuchiol in cosmetic products

**DOI:** 10.1038/s41598-023-41076-7

**Published:** 2023-08-24

**Authors:** Katarzyna Kurpet, Grażyna Chwatko

**Affiliations:** 1 Doctoral School of Exact and Natural Sciences, University of Lodz, 21/23 Jana Matejki Street, 90-237 Lodz, Poland; 2 Department of Environmental Chemistry, Faculty of Chemistry, University of Lodz, 163/165 Pomorska Street, 90-236 Lodz, Poland

**Keywords:** Chemistry, Analytical chemistry

## Abstract

The aim of this study was to develop and validate a simple, fast, and universal reversed-phase high-performance liquid chromatography method with fluorescence detection for the quantitation and evaluation of the stability of bakuchiol in cosmetic products. The analyte was extracted by tetrahydrofuran and separated on a Zorbax Eclipse Plus C18 analytical column (100 × 4.6 mm, 3.5 μm particle size) by a gradient elution program with the mobile phase consisting of water and acetonitrile and a flow rate of 1.0 mL min^−1^. The column temperature was held at 25 °C and fluorescence detection was performed at excitation and emission wavelengths of 264 and 338 nm, respectively. The stability studies of bakuchiol in cosmetic products were conducted under various conditions, including thermal and photolytic degradation, according to International Conference on Harmonization Guidelines. The calibration curve was linear in the range of 0.5–50.0 μg g^−1^ with a correlation coefficient greater than 0.9999. The limits of detection and quantification of the method were 0.1 and 0.5 μg g^−1^, respectively. Recovery values were in the range of 93.37–106.39 μg g^−1^, with relative standard deviations less than 6%. The method has been successfully applied to analyze different types of cosmetic products and proved to be reliable.

## Introduction

Various plants and their extracts have been used in folk medicine for centuries. Currently, the beneficial biological effects of these products, known from traditional use, are confirmed by numerous scientific studies. Among the many active compounds found in plants, there are terpenoids, which include bakuchiol^1–3^. Bakuchiol (4-(3-ethenyl-3,7-dimethyl-1,6-octadienyl)-phenol) is partly phenolic and partly terpene, so it is a meroterpene that combines those two structural elements in a single molecule. Bakuchiol has many beneficial properties for living organisms such as anti-aging, anti-pigmentation, anti-acne, anti-cancer, hepatoprotective, cardioprotective, hypoglycemic, hypolipemic, and antidepressant, antioxidant, anti-inflammatory, antiobesity, and antimicrobial activities, moreover it shows the effect of reducing postmenopausal bone loss^4–9^. Mainly due to the antioxidant, antimicrobial, emulsifying, and skin-improving properties of bakuchiol, it is widely used in cosmetology and dermatology^1,7,10,11^. Recently, also bakuchiol derivatives are being investigated for dermatological use^12^. Bakuchiol was first obtained from the medicinal plant *Psoralea corylifolia*, which is widespread in India, China, and Southeast Asia and used in Chinese and Indian folk medicine^1,5,6^. This plant is still the primary source of bakuchiol. The other plants in which bakuchiol has been detected are *Prosopis glandulosa*, *Otholobium pubescens*, *Pimelea drupacea*, *Ulmus davidiana*, *Piper longum*, *Aerva sanguinolenta*, *Fructus psoraleae*, *Psoralidium tenuiforum*, *Bridelia retusa*, *Elaeagnus bockii*, *Spiraea formosana*, and *Nepeta angustifolia*^6,13,14^. These plants grow in China, Australia, Peru, Sri Lanka, Indonesia, Northern Mexico, and the Northwest United States but mostly in Indochina (Mainland Southeast Asia), so the popular ingredient of the cream, bakuchiol of natural origin, is obtained from Asian plants. *Psoralea corylifolia* and some of the other bakuchiol-containing plants, that have been tested so far, belong mainly to the Fabaceae family, but also *Thymelaeaceae*, *Ulmaceae*, *Piperaceae*, *Phyllanthaceae*, *Elaeagnaceae*, and *Lamiaceae*. To produce cosmetics containing bakuchiol is used bakuchiol carrier oil or about 90% pure bakuchiol which is achieved through an extraction process. Bakuchiol extract purification^4,6,15^ involves several different steps and is not always completely effective. Up to now, a few methods based on high-performance liquid chromatography (HPLC) with various types of detection have been developed for bakuchiol determination in *Psoralea corylifolia*^4,16–22^. Although this technique allows for the easy performance of both qualitative and quantitative analyzes of the ingredients of cosmetic preparations in a relatively short time, there are no published methods that could be applied to the quality control of cosmetics with bakuchiol. According to EU law^23^, the control of the content of active ingredients and the assessment of stability in cosmetics are not required, and the quality of cosmetic products largely depends on the manufacturer. For this reason, the growth of the cosmetic products market is accompanied by a growing concern about their effectiveness on the part of their users. Therefore, it seems extremely important to assess the stability of active compounds in cosmetic products, as it has a direct impact on the quality and safety of cosmetic products. Due to the wide range of positive properties of bakuchiol, more and more companies are trying to add it to their cosmetics, thanks to which the offer on the market is expanding. With the increase in the number of such products, the need for their proper quality control also increases. The present work aimed to develop and validate a new reversed-phase HPLC method with fluorescence detection (FLD) for reliable quantification of bakuchiol in cosmetic products. The stability studies of bakuchiol in cosmetic matrix under various conditions were also carried out. The proposed method was applied to the quality control of different categories of commercial cosmetic products containing bakuchiol with successful results. As far as we know, this is the first HPLC-FLD method for the determination of bakuchiol in cosmetics, as well as the only report about the evaluation of the stability of bakuchiol in these matrices.

## Results and discussion

### Sample preparation

The wide range of products and the intricacy of their formulation are a huge challenge for analytical chemistry. Cosmetic sample matrices typically contain many ingredients, and formulation analysis, particularly the quantification of active ingredients, often requires the development of appropriate sample preparation conditions for analysis. Dissolution of analytes can be carried out with appropriate chemicals, by heating or exposure to ultrasound or microwave radiation. Analytical procedures sometimes require purification or concentration of analytes. Both solid-phase extraction and liquid-liquid extraction are used for this purpose. An efficient extraction procedure is important for the accurate determination of active ingredients in cosmetics. In the present method, we optimized extraction conditions for bakuchiol-containing cosmetic products, including the extraction solvent, the cosmetic weight-to-volume ratio of the extractant, the extraction kinetics, and the temperature of centrifugation.

#### Selection of the extractant

The extraction efficiency of commonly used extraction solvents, including acetonitrile, tetrahydrofuran, methanol, ethanol, and 1,4-dioxane was compared for seven bakuchiol-containing cosmetics from three different categories of cosmetic products. The choice of solvents for extraction yield evaluation was guided by their polarity, selectivity toward the analyte, and miscibility with the mobile phase. The results are shown in Supplementary Fig. 1 and demonstrate that: (a) generally, methanol and ethanol had the poorest extraction rate of bakuchiol from the tested products; (b) the extraction effect of 1,4-dioxane is strong; however, its main disadvantage is its melting point, which is 11.8 °C, making it impossible to continue the procedure at lower temperatures or to store the processed samples in autosampler at 4 °C until analysis; (c) the results indicated that acetonitrile, despite the greatest compatibility with the mobile phase, it does not have sufficient extracting power for most cosmetic products tested, only two products had the highest extraction yield; (d) using tetrahydrofuran the extraction yield was greater than 90% for five of the seven tested cosmetics. Considering the above, tetrahydrofuran was selected as the best extraction solvent.

#### The ratio of the mass of the cosmetic to the volume of the extractant

The amount of required solvent depends largely on the extraction mode used. In the present procedure, the authors wanted to reduce as much as possible the time required to prepare cosmetic samples for analysis, while ensuring efficient quantitative extraction of the analyte, so it was decided to use a single extraction. Therefore, in the next step, the ratio of the sample weight to the solvent volume was selected. For this purpose, 0.5 g of cosmetics was weighed accurately, and then tetrahydrofuran was added in volumes: 100, 200, 300, 400, 500, and 1000 μL. The further procedure was conducted as described in the “Methods” section. The highest recovery of bakuchiol was obtained for the ratio of 1:4 (Supplementary Fig. 2). It is also worth mentioning that the use of tetrahydrofuran in a volume of 200 μl during the extraction step made it possible to inject the same sample multiple times during the subsequent HPLC analysis. Further increasing the volume of the extractant resulted in the sample dilution, as well as, probably, extraction of more cosmetic matrix components and ultimately in the decreased recovery of bakuchiol.

#### Extraction kinetics

The extraction time needed to achieve the highest efficiency was optimized in the range of 2–30 min. The results (Supplementary Fig. 3) revealed that there is no significant influence of this factor on the extraction yield of bakuchiol. The results indicate that an extraction time of 5 min is sufficient for the recovery of bakuchiol from cosmetics with better reproducibility in comparison with two-minute extraction. Therefore, 5 min of extraction was chosen.

#### Centrifugation temperature

During the optimization of the sample preparation step, the influence of the temperature of centrifugation on the peak area of bakuchiol was checked. The following temperatures were selected for evaluation: 4, 10, 15, 25, 30, and 40 °C. The results depicted in Supplementary Fig. 4 show that no significant difference was found between various temperatures of centrifugation according to the peak areas in chromatograms. Nevertheless, the best reproducibility was obtained when the mixture was centrifuged for 10 min at 14,000 rpm and 25 °C. Therefore, the temperature of centrifugation of 25 °C was selected as optimal. In addition, this temperature allows the presented method to be used in other laboratories that do not have centrifuges equipped with thermostats.

#### Extraction efficiency

The extraction efficiency was studied by comparing of peak area of bakuchiol obtained after the analysis of standard solutions with the peak area of analyte obtained after the analysis of the spiked blank samples. The final content of bakuchiol in the samples was 1.0 μg. All the samples were prepared in triplicate. The average extraction yield calculated for several different cosmetic matrices was at the level of 75%.

### Method development

#### Optimization of the fluorescence detection

Bakuchiol contains in its structure fluorophore and can generate native fluorescence without the need for derivatization, so fluorescence detection is expected to be the best choice for the direct determination of this compound in cosmetic products, especially for reliable quality control of new cosmetic formulations, in which the content of bakuchiol can be trace. Since the excitation and emission wavelengths have a direct impact on the sensitivity of fluorescence detection, during method development the spectra of bakuchiol were recorded. To find the optimum excitation and emission wavelengths the stock solution of bakuchiol was diluted to 0.1 μg μL^−1^ by methanol and the fluorescence spectra for this solution were measured. The excitation wavelength from 220 to 324 nm and emission wavelength from 274 to 526 nm were examined. The results (Supplementary Fig. 5) show that the highest sensitivity was achieved under excitation and emission wavelengths of 264 nm and 338 nm, respectively. The excitation wavelength is in accordance with the one obtained by another author^20^, and the difference between emission wavelengths is only 6 nm.

#### Optimization of the chromatographic conditions

Based on the chemical structure and logP (5.09 at 20 °C)^24^ value of bakuchiol that indicate its strong lipophilicity, the RP-HPLC system was selected. For optimization of the chromatographic separation conditions, parameters, such as column type, mobile phase composition, flow rate, elution mode, and oven temperature were examined. Four columns including Agilent Zorbax Eclipse Plus C18 (4.6 × 100 mm, 3.5 μm), Dr. Maisch ReproShell Biphenyl (4.6 × 150 mm, 5 μm), Agilent Zorbax SB-C18 (4.6 × 150 mm, 5 μm), and Thermo Fischer Accucore 150-C4 (4.6 × 150 mm, 2.6 μm) were tested. The Zorbax Eclipse Plus C18 column showed the best performance in terms of good peak separation, peak shape/symmetry, and retention time, so it was used for further studies. The mobile phases used in optimization were methanol and water in different ratios, acetonitrile and water in different ratios, and tetrahydrofuran and water in different ratios. A mobile phase consisted of water (A) and acetonitrile (B) was selected for the determination of bakuchiol. To obtain good resolutions and analysis time, a gradient elution program was chosen. Moreover, different flow rates and column temperatures were evaluated and compared. The results showed that these parameters had little impact on the resolution of the analyte. Therefore, the flow rate of 1.0 ml min^−1^ and the separation temperature of 25 °C were selected. Under the optimum chromatographic conditions, the retention time of bakuchiol is about 6.4 min.

### Method validation

The developed method was validated following the International Council for Harmonization of Technical Requirements for Pharmaceuticals for Human Use (ICH)^25–27^. The validation parameters of specificity, linearity, precision, accuracy, limits of detection (LOD) and quantification (LOQ), carry-over, robustness, system suitability parameters, as well as stability were tested under the optimal separation and detection conditions.

#### Specificity

The ICH guidelines define specificity as the ability to assess unequivocally the analyte in the presence of components that may be expected to be present, such as impurities, degradation products, and matrix components. The specificity of the proposed method is illustrated in Supplementary Fig. 6 where chromatograms obtained from the analysis of a serum sample containing bakuchiol and a blank serum matrix sample are shown. The specificity evaluation showed that no significant response attributable to the interfering components from matrix excipients was observed at the retention time (t_r_) of bakuchiol (t_r_ = 6.433 min). The peak corresponding to the analyte was well-resolved from other peaks in all cases, and no potential interferences affect the accurate determination of the bakuchiol’s concentration.

#### Linearity

The linearity was assessed by the analysis of the spiked cosmetic serum samples at the concentration level of 0.5–50.0 μg g^−1^. The eight-point calibration curve (Supplementary Fig. 7) was prepared using freshly spiked calibration standards in triplicate. Each sample was processed according to the recommended procedure. Test results were evaluated by calculation of a regression line by the method of least squares. The linear calibration range, regression equation, coefficient of determination, limits of detection (LOD) and quantification (LOQ) are listed in Table 1. The coefficient of determination (R^2^ = 1) value demonstrates that there is a strong linear relationship between peak area and the concentration of bakuchiol. The accuracy (expressed as recovery, %) of the back-calculated concentrations of each replicate of the calibration standards tested per concentration level has been within ± 20% of the nominal concentration at the LLOQ and within ± 15% at the other levels. A wide range of linearity covering three orders of magnitude can be considered useful and interesting due to variable concentrations of bakuchiol in cosmetic formulations.

**Table 1:** Validation data.

Matrix	Regression equation	R^2^	Linear range (μg g^−1^)	Precision (%)		Accuracy (%)		LOD (μg g^−1^)	LOQ (μg g^−1^)
				Min	Max	Min	Max		
Cosmetic serum	y = 4825.2x − 466.03	1.0	0.5–50.0	0.32	11.15	95.26	117.41	0.1	0.5

#### Limits of detection and quantification

The LOD and LOQ were established based on measurements of the signal-to-noise ratio of 3 (S/N = 3) or 10 (S/N = 10), respectively. The LOD is 0.1 μg g^−1^ and the LOQ is 0.5 μg g^−1^ which showed a high sensitivity of the proposed chromatographic method.

#### Precision and accuracy

For the quality assessment of the established method, within-run and between-run precision and accuracy were determined using quality control (QC) samples prepared at four concentration levels within the calibration curve range. At each QC concentration level, assays were repeated five times within the same day for within-run precision and accuracy evaluation and during the following three days to determine the between-run precision and accuracy. Precision was expressed as relative standard deviation (RSD, %) while accuracy was determined as percentage recovery of the analyte. Within-run precision range from 2.15 to 5.67% and between-run precision is from 0.79 to 1.78%. Within-run accuracy ranged from 98.77 to 106.39%, while between-run accuracy from 93.37 to 100.92%. The detailed data are presented in Table 2. Obtained results meet the acceptance criteria and indicate that the proposed method is accurate and shows good precision.

**Table 2:** Within-run (n = 5) and between-run (n = 3) precision and accuracy evaluation for bakuchiol in a cosmetic serum sample.

Matrix	Concentration (μg g^−1^)	Precision (%)		Accuracy (%)	
		Within-run	Between-run	Within-run	Between-run
Cosmetic serum	0.5	2.77	0.85	106.33	93.37
	1.5	4.99	1.78	106.39	95.88
	20.0	2.15	1.25	98.77	100.92
	40.0	5.67	0.79	100.30	99.78

#### Carry-over

During the validation of the method, carry-over, defined as a change in the measured concentration due to analyte residue from the previous sample in the analytical instrument, was assessed. For this purpose, blank samples of cosmetic serum were analyzed after the calibration standard with the highest concentration of 50 μg g^−1^ (Supplementary Fig. 8). Analyzes were performed on samples prepared in triplicate. As recommended^26^, carry-over should not be greater than 20%. In the proposed method, the carry-over was 18.34%, which allows the injection of the next study samples immediately after those with the expected high concentration of bakuchiol, without the need to inject blank samples between analyses.

#### System suitability parameters

To maintain the quality and reliability of the analytical measurements, the system suitability parameters were analyzed as part of the validation process to verify that the system was performing according to predetermined criteria and could generate accurate and precise results over time. For this purpose, six replicates of quality control sample of bakuchiol were injected into HPLC system and column performances such as number of theoretical plates (N), capacity factor (k′), resolution (Rs), tailing factor (Tf), and %RSD were evaluated and compared with the defined acceptance criteria^28,29^. High number of theoretical plates (19002.5 ± 187.6) with a low value of %RSD = 0.99 indicate good separation of bakuchiol on the Zorbax Eclipse Plus C18 column. A perfectly symmetrical peak (Gaussian in shape) should have a tailing factor value of 1.0, but typically values should fall between 1.0 and 1.5. A tailing factor greater than 2.0 is unacceptable. Tailing factor for bakuchiol was 1.119 ± 0.008 with %RSD = 0.68, thus the peak was in symmetrical shape. In practical terms, the resolution value above 2.0 is considered acceptable for most analytical purposes in chromatography. A resolution value of 7.582 ± 0.057 (%RSD = 0.75) indicates excellent separation between adjacent peaks with the ability to accurately quantify the bakuchiol in the sample. The capacity factor (5.541 ± 0.047, %RSD = 0.85) suggests that bakuchiol is sufficient retained by the stationary phase and well-suited for the chosen chromatographic conditions and column. It results in improved separation and resolution of the compound. Acceptance criterium for value of k′ is > 2. However, it should be noted that very high retention factors can lead to potential peak broadening, which can affect overall chromatographic performance. Injection precision expressed as %RSD was assessed using the retention time of the analyte and was 0.39% (t_r_ = 6.31 ± 0.02), indicating consistent and accurate injection onto the column. Thus, all system suitability parameters were within the acceptance criteria.

#### Robustness

In the present work, robustness of the developed method was investigated by small alterations of some chromatographic conditions such as a flow rate (± 0.1 ml min^−1^), a separation temperature (± 0.5 °C), and the influence of variation of acetonitrile in mobile phase composition for initial conditions (± 2%). The most important responses considered in the evaluation were retention times, peak areas, and resolutions. After the introduction of small alterations in column temperature, the resolution, peak area, and retention time values were 7.98 ± 0.09 (%RSD = 1.16), 6819.56 ± 68.36 μV s (%RSD = 1.00), and 6.32 ± 0.09 min (%RSD = 1.41), respectively. For the flow rate alterations, these parameters were as follows: 7.59 ± 0.11 (%RSD = 1.46), 6924.78 ± 102.48 μV s (%RSD = 1.48), 6.42 ± 0.10 min (%RSD = 1.50). When the initial concentration of ACN was altered for ± 2%, the resolution, peak area, and retention time values were 7.41 ± 0.10 (%RSD = 1.31), 6774.89 ± 59.93 μV s (%RSD = 0.88), 6.47 ± 0.09 min (%RSD = 1.42), respectively. The values of %RSD < 2 indicate that the method is robust.

### Greenness of the method

**Figure 1:**
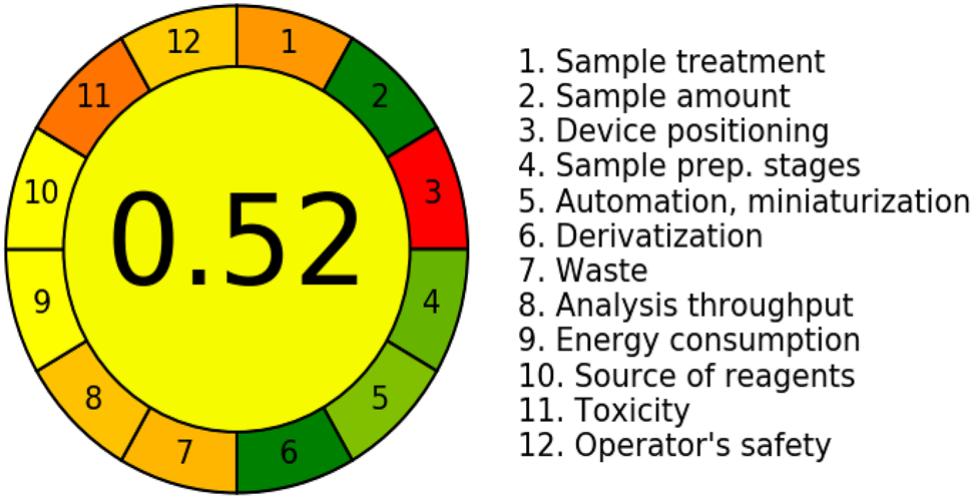
The greenness of the developed method.

When developing analytical methods, it is extremely important to adopt practices that minimize their ecological footprint^30,31^. Applying the principles of green analytical chemistry has many benefits, both for the environment and for the efficiency of laboratory work. Reducing the use of toxic or hazardous chemicals, reducing the use of solvents, and using raw materials more efficiently lead to fewer emissions and less waste generated. Minimizing the consumption of reagents and reducing the number of stages of sample preparation for analysis is also associated with lower water and energy consumption. Optimizing processes according to the principles of green analytical chemistry also translates into fewer health risks for laboratory workers. The introduction of green analytical methods promotes the search for new solutions and technologies, which has recently led to the development of innovative and more efficient ways of analysis. In this study, the environmental impact of the developed method was evaluated using the AGREE—Analytical Greenness Calculator tool^31^. The calculated greenness of the developed chromatographic method is 0.52 (Fig. 1). The procedure consisted of external sample pretreatment and batch analysis with a reduced number of steps, resulting in a score of 0.3 (principle 1). The weight of the cosmetic sample was minimized to 0.05 g, resulting in a maximum score of 1.0 (principle 2). The measurement was performed off-line (principle 3, score equal to 0.0) and the sample preparation procedure consisted of a maximum of 4 steps, such as extraction, centrifugation, filtration, and dilution (principle 4, score equal to 0.8). The method is semi-automatic and sample preparation has been miniaturized (principle 5) giving a score of 0.75. No derivatization step was required (principle 6), resulting in a score of 1.0. Analytical waste consisted of 0.05 g of sample, 2.8 g of consumables, 200 µL of tetrahydrofuran (0.176 g) and 11 ml of HPLC mobile phase including 8 ml (6.29 g) of acetonitrile (principle 7, score equal to 0.36). Only one analyte was determined in a single run, and the sample throughput (samples analyzed per hour) was 6 (principle 8, score of 0.38). In our protocol, the most energy-intensive technique was liquid chromatography (principle 9), resulting in a score of 0.5. In the developed method, we used some bio-based reagents (principle 10, score equal to 0.5). The procedure required 8.2 mL of toxic solvents (principle 11, score of 0.23), which are highly flammable, toxic to aquatic life and can form explosive mixtures in the air (principle 12, score of 0.4).

### Stability studies

A stability assessment was performed to ensure that each step taken during sample preparation and analysis, as well as the storage conditions used for the tested commercial cosmetic products containing bakuchiol, did not affect the concentration of the analyte. The evaluation of the stability of bakuchiol in cosmetic matrix included tests such as freeze-thaw stability, bench-top stability, and long-term stability. These stability data for the spiked cosmetic serum samples at a concentration of 1.5 (low QC) and 40.0 μg g^−1^ (high QC) are presented in Table 3. Based on the results, we can confirm the stability of bakuchiol in the cosmetic matrix since all recovery values at each concentration level were less than ± 5% of the nominal concentration.

**Table 3:** Stability of the bakuchiol in cosmetic serum matrix, n = 3.

Added concentration(μg g^-1^)	Calibration curve equation	R^2^	Found ± SD (μg g^−1^)	Precision (%)	Recovery (%)
Freeze–Thaw stability					
1.5	y = 4493.5x − 993.35	0.9997	1.50 ± 0.01	0.96	99.73
40.0			40.52 ± 0.29	0.71	101.31
Bench-top stability					
1.5	y = 5053.2x − 1677.7	0.9995	1.56 ± 0.01	0.45	103.68
40.0			39.63 ± 0.29	0.72	99.10
Long-term stability (− 20 °C)					
1.5	y = 4205.8 + 493.52	1.0	1.47 ± 0.05	3.17	98.23
40.0			40.22 ± 0.12	0.30	100.55
Long-term stability (− 80 °C)					
1.5	y = 4205.8 + 493.52	1.0	1.54 ± 0.04	2.53	102.40
40.0			40.06 ± 0.59	1.47	100.16

Furthermore, none of the potential degradation products were detected when long-term stability was tested. In the next step, the stability of processed spiked cosmetic samples (extracts) in the autosampler at 4 °C for 12 h was determined. The results (Supplementary Fig. 9) indicate that bakuchiol in extracts is stable under established conditions over the period studied.

The stability of the bakuchiol in stock and working solutions was evaluated under the storage conditions recommended by the manufacturer (− 20 °C). Working solutions were each time prepared in triplicate by appropriate dilution of 10 μg μL^−1^ stock solutions of bakuchiol with methanol to concentrations of 0.005 and 0.5 μg μL^−1^. Stability tests for the stock solution were conducted for one year, while working solutions were analyzed immediately after preparation, after two days, and after two weeks. As the results shown in Table 4 indicate, the back-calculated concentrations of bakuchiol, for both the lowest and highest concentrations of the working solutions, were within ± 1% of the nominal concentration, regardless of the time that had passed since their preparation. Tests conducted confirm the stability of bakuchiol for one year for the stock solution and up to two weeks for working solutions.

**Table 4:** Stability of bakuchiol in stock and working solutions, n = 3.

The fresh stock solution of bakuchiol (10 μg μL^−1^)				The one-year stock solution of bakuchiol (10 μg μL^−1^)			
Analyte concentration (μg μL^−1^)	Found ± SD (μg μL^−1^)	Precision (%)	Recovery (%)	Analyte concentration (μg μL^−1^)	Found ± SD (μg μL^−1^)	Precision (%)	Recovery (%)
Fresh working solutions							
0.005	0.005014 ± 0.00012	2.39	100.27	0.005	0.00499 ± 0.00025	4.92	99.71
0.5	0.50494 ± 0.00741	1.47	100.99	0.5	0.49814 ± 0.00389	0.78	99.63
2 days working solutions							
0.005	0.00501 ± 0.00006	1.10	100.16	0.005	0.00500 ± 0.00015	3.08	100.08
0.5	0.50189 ± 0.00720	1.44	100.38	0.5	0.50235 ± 0.00448	0.89	100.47
2 weeks working solutions							
0.005	0.00496 ± 0.00003	0.61	99.11	0.005	0.00496 ± 0.00011	2.18	99.14
0.5	0.50070 ± 0.00498	0.99	100.14	0.5	0.49963 ± 0.00459	0.92	99.93

In the final step, accelerated stability studies of bakuchiol in a cosmetic matrix under thermal and photolytic degradation conditions were conducted. To this end, we tested the stability of bakuchiol in spiked cosmetic samples that were stored under various test conditions for 24 h. Bearing in mind the different places and conditions for storing cosmetics by customers, the most common are a refrigerator, closed cabinets in the rooms, or a bathroom where the temperature may temporarily increase as a result of taking hot baths or showers, and then stabilize at a predetermined level due to air diffusion, the following temperatures were selected for testing: 4, 25 and 40 °C. All samples were prepared in triplicate and tested at preset temperatures, with artificial light exposure or in darkness. The analyte content in each case was up to ± 5% of the initial content with the RSD values ranging from 0.84 to 3.35% (Table 5). Furthermore, no additional degradation products of the other components of the cosmetic matrix were observed on the chromatograms. The obtained results seem to be satisfactory for customers of cosmetic drugstores who use cosmetics with bakuchiol.

**Table 5:** Results from photostability and thermal degradation studies, n = 3.

Temperature (°C)	Access of light	Found ± SD (μg g^-1^)	Precision (%)	Recovery (%)
Low QC = 1.5 μg g^-1^				
4	Light	1.46 ± 0.02	1.39	97.32
25		1.53 ± 0.03	2.04	101.92
40		1.56 ± 0.02	1.33	104.33
4	Dark	1.53 ± 0.02	1.10	102.05
25		1.53 ± 0.04	2.78	102.31
40		1.54 ± 0.04	2.28	102.35
High QC = 40.0 μg g^-1^				
4	Light	40.04 ± 0.84	2.10	100.09
25		39.61 ± 0.46	1.15	99.03
40		40.93 ± 0.34	0.84	102.32
4	Dark	41.29 ± 1.38	3.35	103.22
25		40.13 ± 0.44	1.10	100.34
40		40.37 ± 0.47	1.16	100.92

### Application to cosmetic products

With the still increasing number of cosmetic products with bakuchiol on the market, there is also a growing need for their proper quality control, which was performed in this study. The developed and validated method was applied to the quality control of various cosmetic products containing bakuchiol purchased at cosmetic drugstores or local supermarkets. The content-related quality control of bakuchiol in different commercial cosmetic products was assessed by comparing the determined and the declared content. In total, eight different bakuchiol-containing cosmetic products from various brands were analyzed. These cosmetics were manufactured from 2021 to 2022. According to the label information of each product, for cosmetics with bakuchiol content over 1% or 2%, a dilution of 1:50 or 1:100, respectively, with the mixture of water and acetonitrile (35:65 v/v) was carried out before HPLC analysis. Concentrations were calculated based on the calibration curve, then recalculated considering 100% extraction efficiency, and ultimately expressed as a weight percentage. The results of the quantitative evaluation of bakuchiol in the tested products are summarized in Table 6, and the obtained chromatograms are shown in Supplementary Fig. 10. The results indicate that the proposed method could be an effective tool for bakuchiol detection in new cosmetic formulations since the content found is in accordance with that declared by manufacturers for three cosmetics with the packaging labeled amount of bakuchiol.

**Table 6:** Analytical data on the determination of bakuchiol in cosmetic products by HPLC-FLD, n = 3.

Cosmetics	Amount in % w/w (mean ± SD)	
	Declared	Found
Cream 1	n.d.*	0.00825 ± 0.00023
Cream 2	n.d	0.00080 ± 0.00002
Cream 3	n.d	0.00028 ± 0.00001
Cream 4	1.0	0.98256 ± 0.02414
Serum 1	n.d	0.01380 ± 0.00082
Serum 2	1.0	1.09818 ± 0.00833
Serum 3	2.0	1.98814 ± 0.01858
Face mask 1	n.d	0.00145 ± 0.00005

*n.d. = no data.

## Methods

### Chemicals and reagents

(S)-Bakuchiol, as well as tetrahydrofuran and acetonitrile of HPLC gradient grade were purchased from Sigma Aldrich (St. Louis, MO, USA). Ethyl alcohol absolut 99.8% and 1,4-dioxane of P.A.-BASIC pure were supplied by POCH (Gliwice, Poland). HPLC-grade methanol was obtained from J.T. Beaker (Deventer, The Netherlands). Deionized water was prepared in our laboratory using the Mili-QRG system (Millipore, Vienna, Austria). The cosmetic products containing bakuchiol, including creams, sera, and face masks were purchased from cosmetic drugstores or local supermarkets. For the validation of the method, an anti-wrinkle, bakuchiol-free serum from the same manufacturer as the tested bakuchiol-containing serum was used as a blank sample.

### Instrumentation

Experiments were performed on an integrated LC-4000 Series JASCO RHPLC system (JASCO, Tokyo, Japan) equipped with a quaternary pump (model No. PU-4180, Tokyo, Japan), a vacuum degasser, an autosampler (model No. AS-4150, Tokyo, Japan), a column oven (model No. CO-4062, Tokyo, Japan), and a fluorescence detector (model No. FP-4020, Tokyo, Japan). The detector operated at the excitation and emission wavelengths of 264 nm and 338 nm, respectively. System control and data acquisition processes were performed using the ChromNAV2 software. Spectra Manager ver. 2 was used to analyze the spectra.

### Chromatographic conditions

The chromatography was performed at 25 °C on a reversed-phase analytical column Zorbax Eclipse Plus C18 (100 × 4.6 mm, 3.5 μm) from Agilent Technologies (Waldbronn, Germany). The mobile phase consisted of water (A) and acetonitrile (B), and it was pumped at a flow rate of 1.0 mL min^−1^. Chromatographic separation was achieved in 10 min with gradient elution profile as follows: 0–2 min, 65% B; 2–3 min, 65-75% B; 3–4 min, 75–80% B, 4–6 min, 80–90% B, 6–8 min, 90–65% B; 8–10 min, 65% B. Fluorescence detector conditions were set as following: response 1.5 s, gain 1×, scan speed 200 nm s^−1^, recorder range—short. The injection volume was 5 µl. The autosampler temperature was set at 4 °C. Prior to chromatographic analysis, all sample solutions were filtered through 0.20 µm nylon syringe filters (Merck, Darmstadt, Germany). Identification of the bakuchiol peak was based on a comparison of retention times and fluorescence spectra, taken at the real-time analysis, with a corresponding set of data obtained for the authentic standard compound.

### Stock and working standard solutions

The stock solution of bakuchiol (10 mg mL^−1^) was prepared by dissolving it in methanol. The solution was stored in the dark at − 20 °C for one year without any noticeable change in the content. Working standard solutions of bakuchiol were freshly prepared by diluting the standard stock solution with methanol as needed.

### Quality control samples and calibration standards

The calibration spiked cosmetic serum samples of bakuchiol were prepared every working day at the concentration levels of 0.5, 0.7, 1.0, 1.5, 2.0, 5.0, 10.0, and 50.0 μg g^−1^. Quality control samples of bakuchiol were prepared at four concentration levels of 0.5 (LLOQ), 1.5 (low QC), 20.0 (medium QC), and 40.0 μg g^−1^ (high QC). The calibration standards and four quality control samples were prepared in triplicate by diluting the stock standard solutions of bakuchiol with methanol and adding the proper aliquots to accurately weighted bakuchiol-free serum to achieve the concentrations as mentioned above.

### Sample preparation

Commercial cosmetic products were accurately weighted (0.05 g) into a 1.5 mL Eppendorf tube, followed by the addition of 200 μL of tetrahydrofuran. The samples were further vortexed (BioSan, MSV-3500 Multi-Speed, Riga, Latvia) for 5 min at 3500 rpm and room temperature. Then, the mixture was centrifuged (Hettich, Mikro 220 R, Tuttlingen, Germany) for 10 min at 14,000 rpm and 25 °C. The extracts from commercial cosmetic samples that contain ≥ 1% or ≥ 2% of bakuchiol (according to the manufacturer’s data) were diluted 1:50 or 1:100 with a mixture of water and acetonitrile (35:65 v/v), respectively. The supernatant was filtered through a nylon syringe filter with a 0.20 μm pore size (Merck, Darmstadt, Germany) and transferred into vials for chromatographic analysis. The extraction efficiency was evaluated by comparing the peak areas.

### Method validation

The method was validated following ICH Guidelines^25–27^ in terms of specificity, linearity, accuracy, precision, the limit of detection, the limit of quantification, system suitability parameters, carry-over, robustness, and stability. The validation parameters mentioned above were estimated under optimal separation and fluorescence detection conditions.

#### Specificity

The specificity of the developed method was evaluated using matrix samples without bakuchiol obtained from various sources (blank samples) and commercial cosmetic products containing the analyte. We compared the chromatograms obtained from the analysis of cosmetics samples and corresponding blank cosmetics matrix samples to demonstrate that there is no response attributable to potential interferences at the retention time of bakuchiol in the blank samples.

#### Linearity

The linearity of the method was evaluated triplicated using eight freshly spiked calibration standards ranging between 0.5 and 50 μg g^−1^. The calibration curve was constructed using a linear least-squares regression model by plotting the average peak area values against the analyte concentrations. Blank samples were not included in the determination of the regression equation for the calibration curve.

#### Limits of detection and quantification

The limits of detection and quantification were evaluated using a signal-to-noise ratio of 3:1 and 10:1, respectively, within a defined region and situated equally around the place where the peak of bakuchiol would be found. Determination of the signal-to-noise ratio was performed by comparing signals from samples with known low concentrations of bakuchiol in the range of 0.1–2.0 μg g^−1^ with those of blank samples. All the samples were prepared and analyzed in triplicate.

#### Precision and accuracy

Assay precision and accuracy were determined within-run and between-run. Within-run precision and accuracy were evaluated by analyzing five replicates of the quality control samples at concentrations 0.5, 1.5, 20.0, and 40.0 μg g^−1^ in each analytical run and on the same day. Between-run precision and accuracy were evaluated by analyzing quality control samples at each above-mentioned concentration level in three analytical runs over three days. The accuracy was expressed as a ratio of the found and added concentration of an analyte (recovery, %), and the precision was determined as a relative standard deviation (RSD, %).

#### Carry-over

The carry-over was assessed by analyzing blank samples after the highest calibration standard of bakuchiol at the concentration level of 50.0 μg g^−1^. The samples were analyzed in triplicate. The carry-over was expressed as a ratio of the mean peak area of bakuchiol in the blank samples following the highest calibration standard and the mean response of the analyte in the calibration standard at the ULOQ.

#### System suitability tests

System suitability parameters were assessed by analyzing six replicates of quality control sample at concentration 40 μg g^−1^ according to the guidelines for analytical method validation^28,29^. The parameters used in the system suitability tests included number of theoretical plates (N), capacity factor (k′), resolution (Rs), tailing factor (Tf), and injection precision. The values of percentage of relative standard deviation (%RSD) for these parameters were estimated. The results of each system suitability test expressed as mean ± SD were compared with the acceptance criteria.

#### Robustness

Robustness of the developed method was studied by deliberate changes of the analytical parameters including flow rate (1.0 ± 0.1 ml min^−1^), column temperature (25 ± 0.5 °C), and concentration of acetonitrile in the mobile phase. The responses considered in the evaluation were retention times, resolutions, and peak areas. Robustness was assessed by analyzing three replicates of quality control sample of bakuchiol at concentration of 1.5 μg g^−1^.

### Greenness of the developed analytical method

The AGREE—Analytical GREEnness Calculator^31^ software (ver. 0.5 beta) was applied to evaluate the greenness of the developed chromatographic method.

### Stability studies

The stability of the bakuchiol in the cosmetic matrix was evaluated by analyzing quality control samples at the concentration level of 1.5 (low QC) and 40.0 μg g^−1^ (high QC). The samples were analyzed at time zero and after the applied conditions that were evaluated. For each stability test, the quality control samples were analyzed against the calibration curve in the range of 0.5–50.0 μg g^−1^. Freeze-thaw stability was assessed after three cycles of freezing at − 20 °C and thawing at ambient temperature with the freezing time of the quality control samples between runs being at least 12 h. The bench-top stability test was conducted as follows: low and high QC were thawed at room temperature and kept on the bench top for the same duration as the commercial cosmetic products with bakuchiol. The long-term stability of bakuchiol in the cosmetic matrix was established at − 20 and − 80 °C for 2 months. The autosampler stability of the bakuchiol in processed samples stored at 4 °C was evaluated for 12 h. Photostability and thermal degradation tests were carried out at 4, 25, and 40 °C for 24 h. The stability of the bakuchiol in stock solution at the concentration level of 10 mg mL^−1^ was determined for 1 year. For evaluation of the stability of the bakuchiol in working solutions, the freshly prepared stock solution and the one-year stock solution were used. Working solutions that were tested for stability were prepared and analyzed at concentration levels of 0.005 and 0.5 μg μL^−1^. Changes in the bakuchiol content in working solutions were evaluated immediately after preparation, after two days, and after two weeks. All the samples for each stability study were prepared in triplicate and analyzed according to the proposed sample preparation protocol.

### Application to commercial cosmetic products

Commercial cosmetic products with bakuchiol were purchased on the Polish market, in cosmetic drugstores or local supermarkets, in 2022. In total, eight samples of three different categories of cosmetic products were analyzed: four creams, three sera, and one face mask. The cosmetic products were stored according to the manufacturers’ information. Samples from the obtained cosmetic products were prepared in triplicate and analyzed according to the proposed method.

### Statistical analysis

All calculations, graphs, and statistical analyses were performed using Microsoft Excel 16.0 (Microsoft Corporation). Each value in the charts represents the mean of three independent measurements with the standard deviations indicated. All the results were presented as the means ± SD of at least three chromatographic runs/replicates of QCs. Linear regression was applied to develop an equation for predicting bakuchiol concentration in cosmetics. Linear least-squares regression was used to calculate the linear relationship between peak ranges and analyte concentrations.

## Conclusions

Bakuchiol is a new ingredient in the dermo-cosmetic market. Numerous scientific studies have shown that bakuchiol has an anti-aging effect. It improves the elasticity and firmness of the skin, reduces discolorations, and unifies skin tone. In addition, it moisturizes and soothes inflammation. It also has antibacterial properties. Bakuchiol is referred to as “plant retinol” because it has properties similar to retinol but does not exhibit irritant effects and is an ideal alternative for pregnant women, nursing mothers, and people with very sensitive skin. Unlike retinol, it can be successfully used in the summer, as it does not have photosensitizing properties. Such valuable properties of bakuchiol are increasingly used by the cosmetics industry. The dynamic development of cosmetic products is associated with the development of analytical techniques, which are currently characterized by high sensitivity, speed, and reliability. The process of analyzing cosmetic samples should be as automated as possible, relatively simple, reproducible, and economical in terms of the use of chemical reagents. In the present work, we developed and validated the first HPLC-FLD procedure for the determination of bakuchiol in commercial cosmetic products. It is also the first reported evaluation of the stability of bakuchiol in a cosmetics matrix under various conditions. The proposed method is universal, specific, linear, accurate, and precise with a run time of 10 min that enables the rapid quantification of bakuchiol in different categories of cosmetics. The performed studies confirmed the presence of bakuchiol in a few testing products at levels declared by manufacturers, which proves that the method can be successfully used for reliable quality control of new cosmetic formulations.

### Supplementary Information

Supplementary Figures.

## Data Availability

The datasets generated during and/or analyzed during the current study are available from the corresponding author upon reasonable request.
