# Supplementary material for: Development of a new chromatographic method for the determination of bakuchiol in cosmetic products
Source: Sci Rep. 2023 Aug 24;13:13893. doi: 10.1038/s41598-023-41076-7 (PMC10449805; doi:10.1038/s41598-023-41076-7)
Supplement: Supplementary file 1 — Supplementary Figures. [file 41598_2023_41076_MOESM1_ESM.docx]

Development of a new chromatographic method for the determination of bakuchiol in cosmetic products

**Supplementary Information**

# Katarzyna Kurpet1,2,*, Grażyna Chwatko2

1University of Lodz, Doctoral School of Exact and Natural Sciences, 21/23 Jana Matejki Street, Lodz, 90-237, Poland

2University of Lodz, Faculty of Chemistry, Department of Environmental Chemistry, 163/165 Pomorska Street, Lodz,
90-236, Poland

*[katarzyna.kurpet@edu.uni.lodz.pl](mailto:katarzyna.kurpet@edu.uni.lodz.pl)

**Supplementary Figure 1.** Comparison of the extraction efficiency of commonly used extraction solvent for commercially available cosmetic products (n = 3).


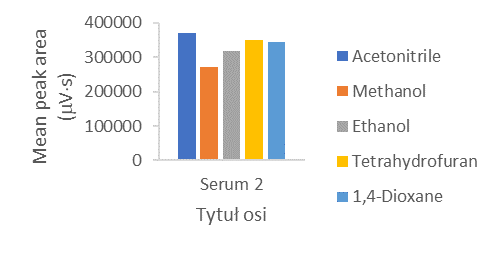

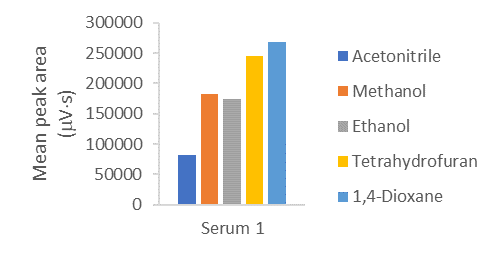

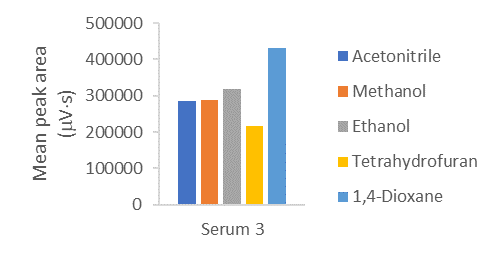

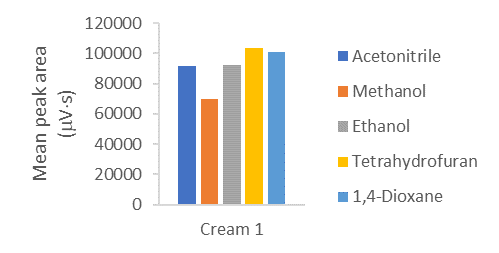

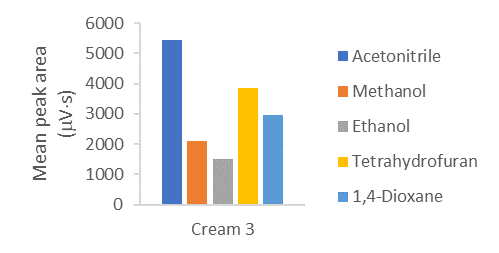

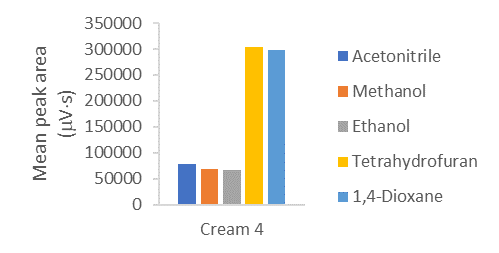

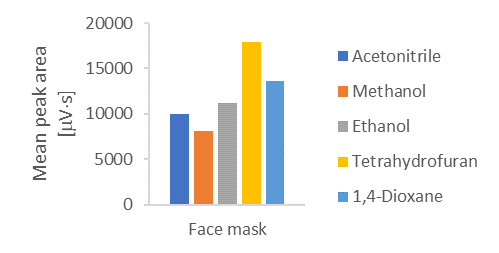

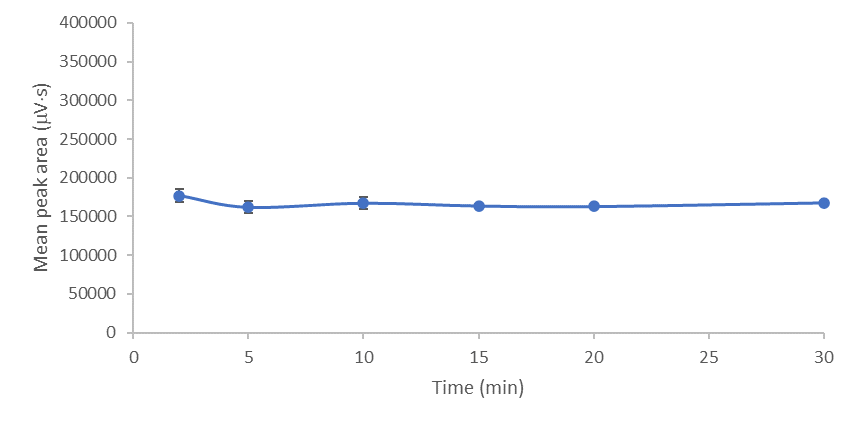


**Supplementary Figure 3.** Effect of extraction time on mean peak area of bakuchiol for serum 1 (n = 3).

**Supplementary Figure 2**. Diagram of the relationship between the average peak area and the ratio of the mass of the cosmetic to the volume of the extractant for serum 1 (n = 3).


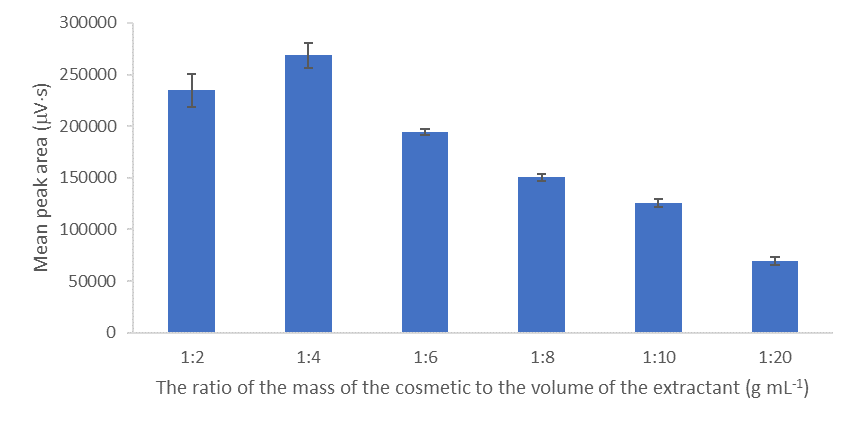

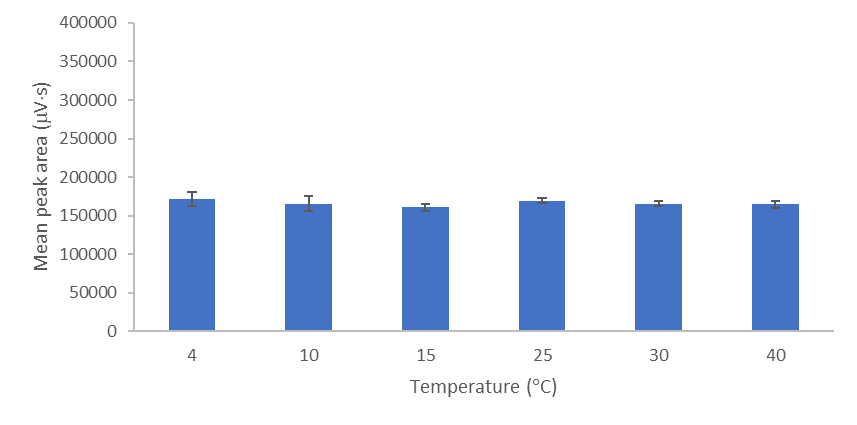


**Supplementary Figure 4.** Influence of centrifugation temperature on average peak area for serum 1 (n = 3).

a

b

**Supplementary Figure 6.** Representative chromatograms obtained from the analysis of (a) a blank serum matrix sample and (b) a serum sample containing bakuchiol.


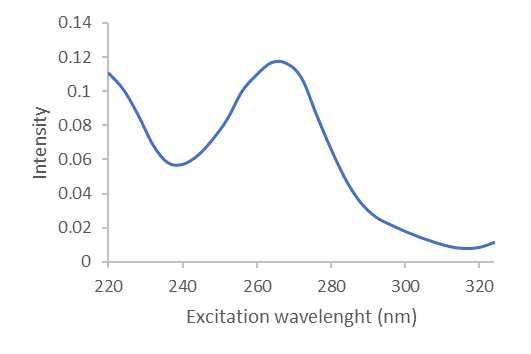

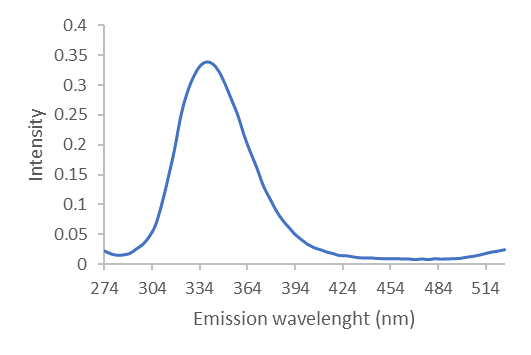


a

b

**Supplementary Figure 5.** Excitation (a) and emission (b) spectra of bakuchiol.


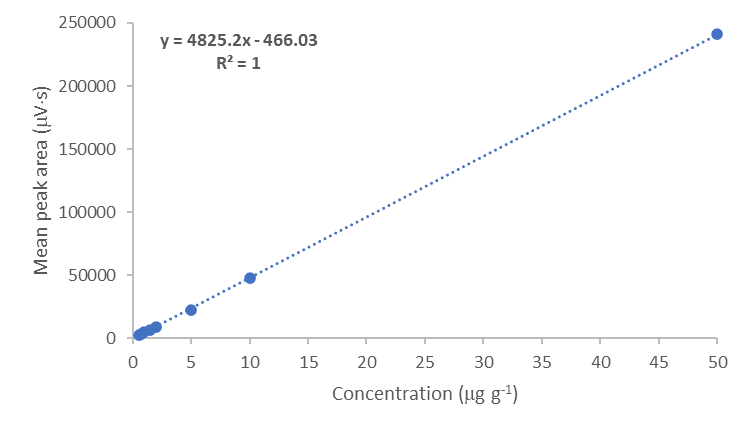


**Supplementary Figure 7.** Calibration curve of spiked serum samples at concentration range of 0.5-50.0 μg g^-1^ of bakuchiol.

**Supplementary Figure 8.** Examples of overlayed chromatograms obtained during carry-over measurements: pink line – blank sample, blue line – the highest calibration standard (ULOQ = 50 μg g^-1^). Retention time of bakuchiol ~ 6.4 min.


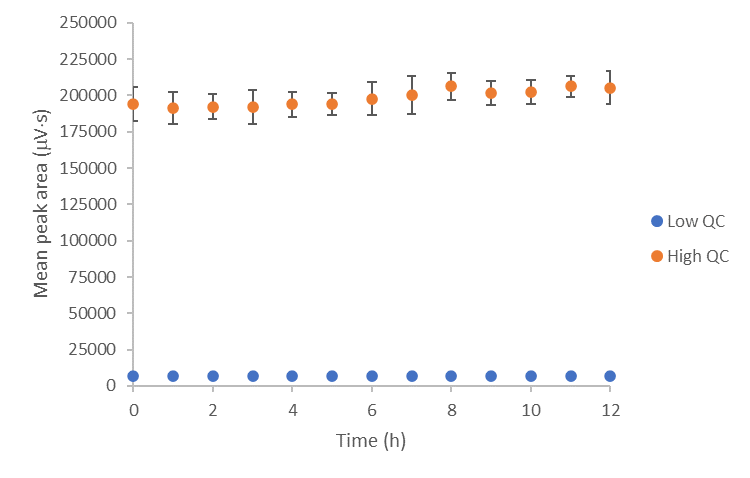


**Supplementary Figure 9.** Stability of the processed cosmetic sample in autosampler at 4 °C.

a

b

c

d

e

f

g

h

**Supplementary Figure 10.** Representative chromatograms obtained from the analysis of various cosmetic products samples after extraction of bakuchiol with tetrahydrofuran: (a) serum 1, (b) cream 1, (c) cream 2, (d) cream 3, (e) face mask, (f) cream 4, (g) serum 2, (h) serum 3. Retention time of bakuchiol ~ 6.4 min.

**bakuchiol**

**bakuchiol**

**bakuchiol**

**bakuchiol**

**bakuchiol**

**bakuchiol**

**bakuchiol**

**bakuchiol**
